# Drivers of adaptive evolution during chronic SARS-CoV-2 infections

**DOI:** 10.1038/s41591-022-01882-4

**Published:** 2022-06-20

**Authors:** Sheri Harari, Maayan Tahor, Natalie Rutsinsky, Suzy Meijer, Danielle Miller, Oryan Henig, Ora Halutz, Katia Levytskyi, Ronen Ben-Ami, Amos Adler, Yael Paran, Adi Stern

**Affiliations:** 1 The Shmunis School of Biomedicine and Cancer Research, Tel Aviv University, Tel Aviv, Israel; 2 Edmond J. Safra Center for Bioinformatics at Tel Aviv University, Tel Aviv, Israel; 3 Department of Infectious Diseases and Epidemiology, Tel Aviv Sourasky Medical Center, Tel Aviv, Israel; 4 Sackler Faculty of Medicine, Tel Aviv University, Tel Aviv, Israel; 5 Clinical Microbiology Laboratory, Tel Aviv Sourasky Medical Center, Tel Aviv, Israel

**Keywords:** Genetic variation, SARS-CoV-2, Genome informatics, Viral infection

## Abstract

In some immunocompromised patients with chronic severe acute respiratory syndrome coronavirus 2 (SARS-CoV-2) infection, considerable adaptive evolution occurs. Some substitutions found in chronic infections are lineage-defining mutations in variants of concern (VOCs), which has led to the hypothesis that VOCs emerged from chronic infections. In this study, we searched for drivers of VOC-like emergence by consolidating sequencing results from a set of 27 chronic infections. Most substitutions in this set reflected lineage-defining VOC mutations; however, a subset of mutations associated with successful global transmission was absent from chronic infections. We further tested the ability to associate antibody evasion mutations with patient-specific and virus-specific features and found that viral rebound is strongly correlated with the emergence of antibody evasion. We found evidence for dynamic polymorphic viral populations in most patients, suggesting that a compromised immune system selects for antibody evasion in particular niches in a patient’s body. We suggest that a tradeoff exists between antibody evasion and transmissibility and that extensive monitoring of chronic infections is necessary to further understanding of VOC emergence.

## Abstract (web summary)

Analysis of mutations that arise in chronic SARS-CoV-2 infections shows both overlap and differences with mutations present in pandemic viral variants of concern, highlighting their distinct drivers of evolution.

## Main

SARS-CoV-2 infections normally resolve clinically within a few days, and RNA shedding can last a few days to a few weeks^1^. However, more and more case reports are accumulating that document chronic infections spanning weeks to many months of infection^2,3^. Notably, chronic infection should not be confused with ‘long COVID’, where infection is cleared rapidly yet symptoms persist^4^; in cases of chronic infections, replicative virus is detected for extended periods of time. To date, all cases of chronic SARS-CoV-2 infection have been associated with a severely immunosuppressed condition, including primary immunodeficiencies, immunosuppressive therapy after solid organ transplants, acquired immunodeficiency syndrome (AIDS) and a compromised immune response associated with hematological cancer and/or its treatment. Presumably, these immune system disorders prevent clearance of the virus as compared to patients with an intact immune system, and, thus, the virus persists for lengthy periods of time, most likely due to the compromised function of the adaptive immune system in these patients^5^.

Longitudinal sequencing of some cases of chronic infection has uncovered striking mutational patterns of evolution and revealed that the rate of evolution is much higher than that observed along transmission chains of acutely infected individuals (for example, refs. ^6–8^). Indeed, several recent reports found that the intra-host variation during acute infections is quite limited^9–12^. Moreover, the transmission bottleneck size was inferred to be quite low in these studies, as evidenced by little to no shared genetic diversity in transmission pairs. Overall, this suggests that adaptive evolution may be limited during acute infections and that most of the diversity observed during SARS-CoV-2 circulation in the human population is due to neutral mutations fixed during the small transmission bottleneck. The low genetic diversity observed in acute infections contrasts with the high genetic diversity observed in the rare cases of chronic infections.

Much interest has arisen in cases of chronic infection since the first emergence of VOCs. One major hypothesis that was proposed when the Alpha VOC was first discovered, and, more recently, with the rise of the Omicron variant, was that these variants evolved due to selection pressure created during chronic infections^2,13^. More specifically, the hypothesis posited that strong selection for antibody evasion mutations may occur in immunocompromised individuals with chronic infection who are treated with convalescent plasma (CP) or monoclonal antibodies (mAbs), collectively denoted here as antibody-based treatments (ABTs).

However, there is no consistent pattern that describes the evolution of SARS-CoV-2 across all chronic infections. Whereas some cases display considerable evolution in the spike (S) protein, in other chronic infections, relatively limited evolution is observed. In this study, we set out to consolidate the evolutionary patterns found across chronic infections by re-analyzing previous reports and by sequencing a cohort of six patients with chronic SARS-CoV-2 infection from the Tel Aviv Sourasky Medical Center (TASMC)^14,15^. We explore the shared and unique mutational patterns that emerge in different chronically infected patients and compare them to those observed in global data reflecting transmission chains in acutely infected individuals. We focus on correlates of adaptive evolution and the potential for the creation of new VOCs.

## Results

### Diverse evolutionary patterns in chronic infections

We begin by defining criteria for a chronic infection. In clinical settings, a chronic infection is often defined as one with both prolonged shedding of viral RNA and evidence of infectious virus, either through virus isolation in tissue culture or via detection of subgenomic RNA. However, when surveying various studies reporting chronic infection, we noted a lack of standardization, with different studies defining chronic infections somewhat inconsistently. Hence, we expanded our focus to include patients displaying high-viral-load (VL) shedding for 20 or more days while mining the literature for all such cases that were accompanied by longitudinal whole-genome sequencing of the virus (Methods). The criterion of 20 days was based on a meta-analysis of the duration of viral shedding (defined as a positive nasopharyngeal polymerase chain reaction (PCR) test) across thousands of patients diagnosed until June 2020, which revealed that mean duration of upper respiratory tract shedding was around 17 days, with a 95% confidence interval ranging from 15.5 days to 18 days^16^. Of note, shedding of replication-competent virus lasted markedly less than 20 days. Moreover, estimates of viral shedding are different in some of the more recently detected SARS-CoV-2 variants, such as Delta and Omicron^17,18^, yet, as described below, our analysis focused on variants that were found in earlier stages of the pandemic.

Our search yielded a total of 21 case reports, all of which reported patients who were diagnosed during 2020 or early 2021, and all of which reported patients who were infected with viruses belonging to lineages that pre-dated the Alpha variant (Supplementary Table 2). In addition, six patients adhering to the above criteria were identified in TASMC, and all available samples were sequenced (Methods). Five TASMC patients suffered from hematologic cancers. The sixth patient suffered from an autoimmune disorder and was treated with a high dose of steroids. The six TASMC patients were all diagnosed in late 2020 or early 2021, with four patients infected with a virus from pre-Alpha lineages and two patients infected with a virus from the Alpha lineage (Supplementary Table 2).

Of the 27 chronically infected patients (mean age (s.d.) 55 (21.3) years; 17/27 male), we inferred that all were immunocompromised due to one or more of the following: hematologic cancer (that inherently tends to lead to immunosuppression), direct anti-B cell treatment, high-dosage steroid treatment or very low CD4^+^ T cell counts (due to AIDS). We observed very different evolutionary outcomes across the range of patients examined, from considerable evolution and antibody evasion observed in some patients to relatively static evolution in others (Table 1 and Supplementary Tables 1 and 2).

**Table 1:** Summary of all 27 patients with chronic SARS-CoV-2 infections

Background condition	Anti-B cell background treatment or inferred B cell depletion	High-dosage steroid treatment	Antibody-based COVID-19 treatment^a^	Days of infection (*n*)^b^	S amino acid replacements^c^	Ref.
Chronic lymphocytic leukemia (CLL)	None	None	CP	105	**∆141-144**	^49^
Lymphoma	CD20 bispecific antibodies	Prednisone	CP	106	None	^50^
B cell lymphoma	CD20 bispecific antibodies	Corticosteroids	None	171	V3G, ∆18-30, S50L, N87S, **∆141-145**, A222V	^6^
Kidney transplant	None	Prednisone	CP	27	**∆141-144, E484K**	^51^
Severe antiphospholipid syndrome	Rituximab	Prednisone	mAb	152	P9L, ∆12-18, ∆141-143, **Y144**-, Y144F, Q183H, **N440D**, T478K, **E484K**, **E484Q**, **F486I**, **Y489H**, Q493K, **S494P**, **N501Y**, I870V, A1020S	^8^
Renal disease	Rituximab	Prednisone	None	16	**E484K, E484Q**	^48^
AIDS	None	None	mAb, CP	23	**E484K**, Q954L	^48^
Follicular lymphoma	Obinutuzumab	None	mAb	89	**E484K**	^48^
Heart transplant	None	Prednisolone	None	27	**E484K**	^48^
CLL	None	None	mAb	72	G1219C, **E484K**	^48^
Kidney transplant	None	Prednisolone	mAb, CP	20	None	^48^
AIDS	None	Dexamethasone	None	190	**E484K**, A1078V, R190K, **K417T**, **F490S**, D427Y, **N501Y**, P9L	^33^
Marginal B cell lymphoma	Rituximab	Prednisolone	CP	101	**∆69-70**, **D796H**, Y200H, T240I, S13I, W64G, P330S, P812S	^7^
Acute lymphoblastic leukemia (ALL)	Inotuzumab	Dexamethasone	None	97	**∆144**, S494P	^52^
Follicular lymphoma	Rituximab	Dexamethasone	None	189	S172A	^34^
Follicular lymphoma	Rituximab	Dexamethasone	None	143	D88E, I788V	^34^
Follicular lymphoma	Rituximab	Methyl-prednisolone	HP	66	None	^34^
AIDS, leukoencephalopathy	None	None	None	75	None	^53^
Heart transplanted, chronic kidney disease	None	Prednisone	None	103	None	^53^
ALL	Low CD19	None	CP	144	T95I, **∆145**, ∆141-144, T22I	^54^
ALL	Low CD19	Dexamethasone	None	162	I197T, S13I, K97M, ∆141-143, R190K, V483A, **E484K**, N211K, N440K	^54^
CLL	Rituximab	None	None	56	**E484Q**	P1
Follicular lymphoma	Obinutuzumab	None	None	65	None	P2
Hodgkinʼs lymphoma	None	None	mAb	88	**G485R**, W258C	P3
Autoimmune skin disease	None	Prednisone	None	36	None	P4
ALL	Inotuzumab	None	CP	75	**E484K, F490L, ∆144**	P5
CLL	None	None	None	37	None	P6

^a^HP = Hyperimmune plasma. Treatments are listed only if they were up to 8 days before sequencing.

^b^Shown is the maximal days-since-infection sequenced.

^c^S amino acid replacements are sorted by order of emergence and are shown in bold when evidence for antibody evasion is available (Methods and Supplementary Table 1). Only mutations reaching a frequency higher than 80% at a given timepoint are shown.

### Evolution in chronic infections versus global transmission chains

**Fig. 1:**
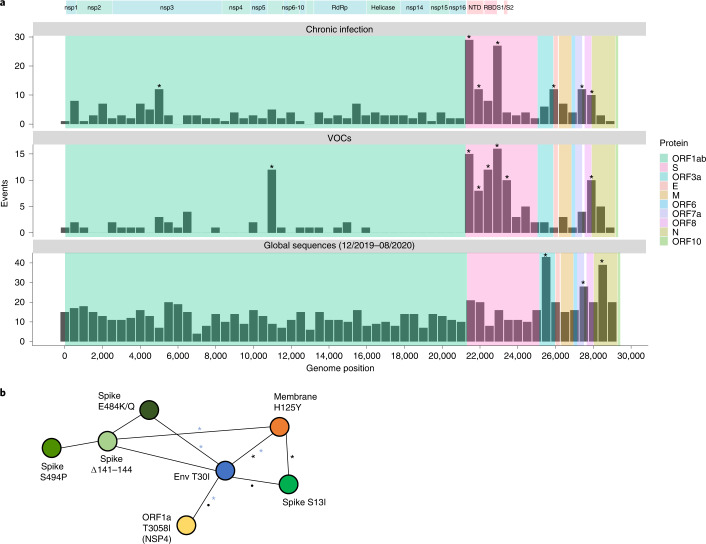
Substitutions in SARS-CoV-2 observed in chronically infected patients and comparison to sequences of circulating viruses. **a**, Comparison of substitutions observed in chronic infections to VOC LDMs and to substitutions dominated by genetic drift during globally dispersed acute infections. Shown are the number of substitutions observed along the SARS-CoV-2 genome, in bins of 500 nucleotides. The upper panel displays substitutions observed at any timepoint of the 27 chronic infections. The middle panel displays LDMs of the five currently recognized VOCs. The lower panel displays substitutions observed globally during the first 9 months of the pandemic, mostly before the emergence of VOCs. Asterisks mark bins enriched for more substitutions using a one-tailed binominal test, after correction for multiple testing (*P* < 0.05; Methods and Supplementary Table 8). The genomic positions are based on the Wuhan-Hu-1 reference genome (GenBank ID NC_045512), and the banner on the top shows a breakdown of ORF1a/b into individual proteins and domains of the S protein (see main text). **b**, A network of co-occurring substitutions across patients with chronic SARS-CoV-2 infection. Each colored circle represents a locus, and a black asterisk and dot represent a significant enrichment under a one-tailed Fisher’s exact test with *P* < 0.05 and *P* < 0.1, respectively, after correction for multiple testing. Blue asterisks represent enrichment of co-occurring substitutions in globally observed sequences using a one-tailed *X*^2^ test, with *P* < 0.05 and *P* < 0.1, respectively, after correction for multiple testing (Methods).

We searched for patterns of evolution across all 27 patients with chronic infection and compared this pattern to the pattern observed under (1) mostly neutral evolution, in the first approximately 9 months of viral circulation^19,20^ (data were obtained from a sample of ~3,500 sequences generated by NextStrain https://nextstrain.org/^21^ (Methods)) and under (2) presumed positive selection, which occurred in the lineages leading to the five currently defined VOCs (Alpha, Beta, Gamma, Delta and Omicron) (data on lineage-defining mutations (LDMs) of VOCs were obtained from https://covariants.org (Fig. 1a and Supplementary Table 4)). In each scenario, we searched for bins—that is, consecutive regions of 500 bases—enriched for mutations (*P* < 0.05, binomial test, after correction for multiple testing; Methods).

During the first 9 months of virus circulation, we noted that 61% of substitutions were non-synonymous, which is generally what we could expect under lack of both positive and purifying selection and in line with reports suggesting incomplete purifying selection during the early stages of SARS-CoV-2 spread^22^. During this time, we observed a relatively uniform distribution of substitutions across most of the genome, with some enrichment in ORF3a, ORF7a, ORF8 and N. This enrichment was previously reported and may be due to more relaxed purifying selection in these regions or higher mutation rates^19^; adaptive evolution at these regions also cannot be ruled out.

In general, the patterns obtained in chronic infections and in the LDMs of VOCs were very similar. The average proportion of non-synonymous substitutions in chronic infections and LDMs of VOCs was 78% and 82%, respectively, which was much higher than that observed during the first stage of the pandemic and generally suggestive of positive selection. On the other hand, we see less similarity between mutations in chronic infections and mutations that fix after a VOC has emerged (Supplementary Fig. 1), with a much lower proportion of non-synonymous substitutions in the latter (on average, 61%). A likely explanation for this observation is that after a VOC spreads in the population, selection is more limited due to the very tight transmission bottleneck^9–12^.

The most striking similarity between chronic infections and VOC LDMs was observed along the S protein and, in particular, at the regions that correspond to the N-terminal domain (NTD) (genomic nucleotides 21,598–22,472) and the receptor-binding domain (RBD) (genomic nucleotides 22,517–23,183). Several mutations at the RBD have been shown to enhance affinity to the ACE2 receptor and allow for better replication^23,24^, whereas other mutations, both at RBD and NTD, are known to enhance antibody evasion^25–27^. The most commonly observed substitutions in chronic infections were in the S protein: E484K/Q and various deletions in the region spanning the NTD supersite, particularly amino acids 140–145, all shown previously to confer antibody evasion^28^. Chronic infections shared the enrichment of ORF3a/ORF7a/ORF8 mutations with the ‘neutral’ set but lacked an enrichment across most of the N protein. Overall, it seems that mutations in chronic infections are predictive of LDMs of VOCs, as was noted previously^2^.

When focusing on the differences between VOCs and viruses in chronic infections, several intriguing differences emerged. First, four VOCs bear a three-amino-acid deletion in the nsp6 protein (ORF1a:∆3,675–3,677), which is an event not observed in our set of chronic infections. Next, in VOCs, there is an enrichment in the region of the S encompassing the S1/S2 boundary (positions 23,500–24,000 in Fig. 1a). This enrichment is primarily driven by S:P681H/R, a highly recurrent globally occurring mutation^29^, surprisingly never observed in our chronic infection set. A recent study analyzed recurrent mutations, with recurrence indicative of positive selection, and tested which of the recurrent mutations led to clade expansion—that is, were associated with onwards transmission^30^. Some recurrent mutations led to more dense clades, suggesting that they were especially successful in driving transmission, whereas others did not lead to considerable onwards transmission, suggesting that they were less successful. Notably, we observed that successful recurrent mutations were almost never present in our chronic set, whereas less successful recurrent mutations (S:E484K/Q and S:∆144) were the most abundant (Table 2). Overall, these results suggest that there may be a tradeoff between antibody evasion and transmissibility. This tradeoff, if it exists, might not play a role in chronic infections but would affect the ability of a variant created in a chronic infection to be transmitted onwards. Thus, only under specific conditions, a transmissible variant would emerge in chronic infections. Four of five VOCs independently acquired a mutation at or near the S1/S2 boundary (S:P681H/R or H655Y), suggesting that this may be a factor driving transmissibility. We note that Beta is an exception with no such mutations, yet this variant also displayed limited global transmission.

**Table 2:** Recurrent mutations observed along the SARS-CoV-2 phylogeny

Protein	Mutation	Clade success	Times observed in chronic infections (*n*)
ORF1a	T3255I	+	1
ORF1a	S3675-,G3677-,F3678-	+	0
S	L18F	+	0^a^
S	T95I	+	1
S	L452R	+	0
S	N501Y	+	2
S	P681R	+	0
N	P199L	+	0
ORF1a	L3606F	–	1
S	H69-,V70-	–	1
S	Y144-	–	7^b^
S	E484K	–	10^c^
S	P681H	–	0
N	S194L	–	0
N	T205I	–	0
N	M234I	–	0

High clade success or low clade success is reported based on measurements of clade logistic growth, with + or – representing higher or lower than average growth, respectively^30^. The last column marks the number of patients in the set of chronic infections herein where a substitution was observed.

^a^Two deletion events were observed at this locus.

^b^An additional two deletion events were observed at loci 141–143.

^c^In three additional patients, E484Q was observed.

We went on to examine co-occurring substitutions, defined as pairs of substitutions that appeared in two or more patients. We used Fisher’s exact test to assess whether pairs of substitutions occurred together more often than expected from their individual frequencies (Methods) as a measure of possible epistasis. Intriguingly, four pairs of substitutions across four different proteins emerged as significantly enriched and formed a network of interactions: T30I in envelope, H125Y in the membrane glycoprotein, S13I in the S protein and T3058I in ORF1a (Fig. 1b). This finding was intriguing on multiple fronts. First, envelope and membrane glycoprotein have generally remained very conserved throughout the entire pandemic, and, specifically, the two replacements found are at highly conserved sites (Supplementary Table 1). However, despite their rarity, we found that some of the pairs of mutations also tend to significantly co-occur in globally dispersed sequences (blue asterisks in Fig. 1b). The replacements in S and ORF1a, on the other hand, have been observed only a small number of times in the global phylogeny. Notably, all of the first three proteins form a part in the virion structure itself; however, the functional meaning of this remains unclear. Other pairs of mutations found to co-occur were the three most common S antibody evasion mutations, yet these co-occurrences were not statistically significant. Larger cohorts of patients and further data will be required to determine the implications of these findings.

### Correlates of antibody evasion

**Fig. 2:**
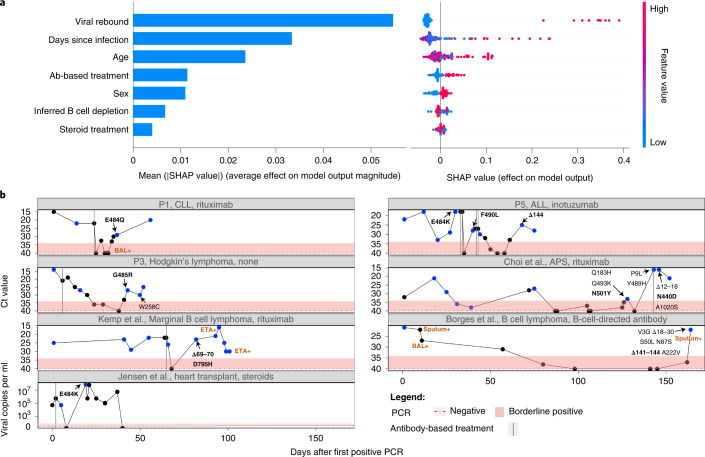
Viral rebound is associated with antibody evasion. **a**, Results of a random forest classifier used to explain an outcome of antibody evasion. The effect of each feature on model outcome is shown: mean SHAP absolute values (left) and individual SHAP values for each feature, ordered based on contribution (right). The color range corresponds to the values of each feature, from red (high value) to blue (low value). **b**, Illustration of individuals who experienced viral rebound and mutations associated with antibody evasion. Ct values are used here as an inversed proxy for VL and are presented according to the day of infection (denoted as number of days after the first positive PCR test), with the dashed red horizontal line and shaded area representing a negative or borderline result, respectively. Blue dots represent samples that were sequenced. Only amino acid replacements in the S protein are shown, with predicted antibody evasion mutations shown in bold (Supplementary Table 1). Positive samples from BAL, ETA or sputum are indicated in brown. Antibody-based anti-COVID-19 treatments are represented by dashed vertical lines on the day of administration. ALL, acute lymphoblastic leukemia; APS, antiphospholipid syndrome; CLL, chronic lymphocytic leukemia; ETA, endotracheal aspirates; P, patient.

We noted very wide variation in the background and treatments given to different patients, both for their background condition and for Coronavirus Disease 2019 (COVID-19). When examining medical background, the patients could be roughly classified into one of the following categories: hematologic cancers, HIV/AIDS, organ transplantation and autoimmune disorders (Table 1). The latter two categories were often treated with steroids. Some, but not all, of the patients with hematological cancer and others were treated with antibodies targeting B cells, presumably causing profound B cell depletion. In line with this, most of the patients with confirmed B cell depletion showed negative serology for SARS-CoV-2 at one or more timepoints (Supplementary Table 1). Some patients were treated with ABT against SARS-CoV-2, whereas others were not; and, in some ABT-treated patients, antibody evasion mutations were detected, whereas, in others, they were not. Finally, we found that, whereas in some ABT-treated patients, antibody evasion mutations were detected, sometimes these mutations fixed before the treatment. The course of VL across time, coupled with ABT, is illustrated for some patients in Fig. 2b. Thus, for example, patient P5 and the patient described by Choi et al.^8^ are shown to fix antibody evasion mutations just before ABT.

We noted that many patients (four of the six patients sequenced herein and several others in the total set of 27 patients) displayed an intriguing cycling pattern of VL (reflected by cycle threshold (Ct) values), with very high Ct values reaching negative or borderline-negative results at one or more stages of the infection, followed by rebound of the virus (Fig. 2b). In the four above-mentioned patients, this rebound was accompanied by clinical evidence of disease, which is highly suggestive of active viral replication. Several different hypotheses could explain this pattern. First, the virus may have cleared and been followed by re-infection with another variant. Because this pattern can be ruled out using sequencing, such cases were excluded from our analysis (Methods). Second, the virus may cycle between different niches, such as upper and lower airways. Its re-emergence in the upper airways (nasopharynx) may be due to selective forces or genetic drift. When considering selective forces, viral rebound may occur due to the near clearance of the virus, driven either by ABT or by the endogenous immune system, and followed by the emergence of a more fit variant with antibody evasion properties.

We fit a random forest classifier to assess the effect of different clinical and demographic features on an outcome of antibody evasion (Methods and Supplementary Tables 2 and 3). We treated each sequencing timepoint as a sample and used age, sex, B cell depletion, steroid treatment, days-since-infection, ABT and viral rebound as explaining variables. We then trained a classifier while considering the structure of the data, composed of samples belonging to the same patient (Methods). After training, we generated SHapley Additive exPlanations (SHAP) values^31,32^ that quantified the effect of each feature on the classifier’s outcome. We found that the feature with the strongest association with antibody evasion was viral rebound, followed by days-since-infection and age (Fig. 2a). Other features had a relatively minor effect, and similar results were obtained with other classifiers (Supplementary Figs. 2 and 3). Regarding the effect of age, we note that young individuals are a minority in this dataset and rarely present an antibody evasion mutation, and, thus, the small sample size may be responsible for the small effect observed with this feature. All in all, these results suggest that ABT is not necessary for driving antibody evasion, in line with the fact that evasion is sometimes observed before (for example, E484K in P5; Fig. 2b) or in the absence of ABT (for example, ref. ^33^). If so, what may be driving immune escape in some patients is actually the weakened immune system of the patient, although ABT and its waning may also play a role in some patients. To summarize, viral rebound may serve as an indicator for the emergence of a mutant with properties of antibody evasion (Fig. 2b), and monitoring for viral rebound in patients with chronic disease is critical.

**Fig. 3:**
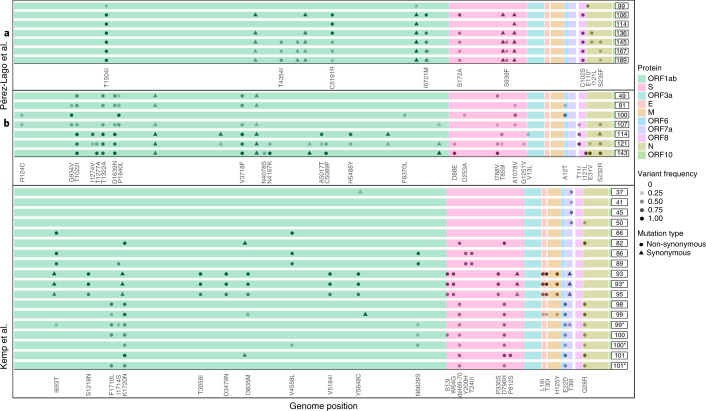
Illustration of polymorphic populations observed across patients. Each series of boxed lines represents a patient, and each line represents a sequenced timepoint with time-since-infection on the right. The different open reading frames are color-coded. For each patient, only mutations relative to the first timepoint sequenced that appeared at a frequency ranging from 20% to 100% are shown. Most samples were nasopharyngeal, except those marked by asterisks, which were obtained from endotracheal aspirates.

Next, we went on to examine patterns of variation over time across the different patients. In many of the case reports, the authors noted the emergence and disappearance (and sometimes re-emergence) of particular substitutions (Fig. 3). For example, in patient B reported by Perez-Lago et al.^34^, the mutation S:A1078V is present at a low frequency on day 81, rises to fixation on day 100 and then drops and disappears from day 107 onwards (Fig. 3). When re-analyzing the data, we noted that this pattern of dynamic polymorphisms across time was observed in most patients (Supplementary Table 2). From an evolutionary point of view, it is quite unlikely for one or more substitutions to disappear from a given population, and, because we observe this at very different loci across all patients, we consider that it is not likely that all of this pattern is due to recurrent sequencing problems or due to biases of the viral polymerase. We and others have previously noted sequencing errors that occur predominantly when VL is low, when errors that occur during reverse transcription or early PCR cycles are carried over to higher frequencies^10,11,35^. However, this phenomenon most often leads to errors in intra-host variants segregating at relatively low frequency and is less common at the consensus sequence level, which is defined here as mutations present at a frequency of 80% or higher. We, thus, conclude that the existence of dynamic polymorphisms likely reflects subpopulations of the virus that co-exist in a patient’s body, as further discussed below.

## Discussion

We performed a comparative evolution study that compares the intra-host dynamics of SARS-CoV-2 as it evolves across different chronic infections and between chronic infections and global transmission chains. We found that viral rebound is associated with antibody evasion mutations and revealed highly polymorphic viral population in many patients. We showed that the overall patterns of mutations observed in chronic infections closely mirror the pattern observed in VOCs, with some notable exceptions. We suggest that most variants emerging in chronically infected patients lack the potential for substantial onwards transmission, possibly due to an absence of key mutations.

We begin by discussing our results on the intra-host evolution of the virus in the chronic infections. We suggest that the most likely explanation for the highly dynamic polymorphisms observed across many infections is the existence of distinct populations residing in different niches, which may correspond to different infected organs, or niches within an infected organ. This is nicely demonstrated by the patient described by Kemp et al.^7^, where both upper and lower airway samples were obtained on the same day (days 99, 100 and 101), revealing different substitutions present in each (Fig. 3). We further suggest that viral rebound coupled with antibody evasion may be enabled by partial penetration of antibodies to a specific niche. Accordingly, this partial penetration will prevent viral clearance on one hand but, on the other, will promote selection for an antibody evasion mutation. Notably, this is supported by the case of patient P1 in this study: during the time a negative nasopharyngeal swab was obtained, a bronchoalveolar lavage (BAL) sample returned as positive, just before the emergence of the E484Q antibody evasion mutation (Fig. 2b). This suggests that the virus continued to thrive in lungs, possibly as a variant adapted to lower airways or as the original nasopharyngeal variant that migrated to the lungs. Alternatively, it is also possible that the immune evasion mutants continued to replicate in the nasopharyngeal swabs but were not picked up by the sample for PCR.

At the outset of this study, we searched for evidence of onwards transmission from a chronically infected patient in our studied cohort, yet we found no such evidence in the publications or from epidemiological investigations of our TASMC patients. This is not necessarily surprising, as often such patients are bedridden and in strict isolation both due to COVID-19 and to their background conditions. However, this does raise the possibility that (1) chronic infections harbor variants that are non-infectious and (2) chronic infections harbor non-transmissible virus—options that we discuss below.

We first consider the option that chronic infections harbor non-infectious virus. In 12 of 27 patients, evidence for viral replication was obtained in the form of culturable virus or in the form of PCR positive for subgenomic RNA. In an additional eight patients, we noted either consistently high VLs (Ct values around 20 or lower) or a negative difference of ten cycles between one PCR outcome and a later one (Supplementary Table 6). In other words, in the latter scenario, VL increased substantially across time in these patients, strongly suggestive of active viral replication. In line with this, we did not find evidence for mutations associated with defective virus, such as premature stop codons, frameshifts or marked deletions outside of the S protein (although in-frame deletions were noted in two patients, in ORF1a; Supplementary Table 1). Thus, to summarize, although we cannot completely rule out the presence of non-infectious, defective virus in chronic infections, we consider it unlikely that all virus is non-infectious in most of the cases examined herein.

We next consider the probability that variants found in chronic infections are transmissible. In most of our patients (15/27), we observed either S:E484K/Q and/or deletions at S:Y144, both of which are highly recurrent mutations in the global phylogeny. However, globally circulating variants bearing these mutations were inferred to be less transmissible than variants bearing other highly recurrent mutations (Table 2 and ref. ^30^). On the other hand, several mutations associated with higher transmissibility (for example, S:P681R and ORF1a:∆3,675–3,677)^30^ were never observed in the chronic infections herein (Table 2).

We note that we do not anticipate selection for mutations enhancing transmissibility in chronically infected patients but, rather, expect selection to promote mutations that enhance viral replication (with immune evasion included in this latter category). However, there is often a link between better net viral replication and higher transmissibility^36–38^. For example, several reports have linked mutations at S:P681 to efficiency of S cleavage, fusion, syncytia formation and higher net VL^38–42^, which theoretically means that this mutation should be selected for also in chronic infections. One possibility is that the sample size of patients in this study was too small. Returning to the example of S:P681H/R, another possibility is that mutations at this locus are under weaker positive selection in the conditions particular to immunocompromised patients. Accordingly, perhaps viral replication in patients with chronic disease tends to occur more prominently in lower airways, with less selection for mutations that enhance replication in upper airways. A recent study has suggested that part of the evolutionary advantage of S:P681H is its ability to evade the interferon (IFN) response of the cell^43^. Many immunocompromised patients are treated with steroids that may alter the IFN response^44,45^; alternatively, the IFN response of immunocompromised patients is also likely weakened. Thus, perhaps in chronically infected immunocompromised patients, selection operates differently than in acutely infected patients, leading to the fact that some mutations observed globally are not observed in these patients.

Finally, an additional possibility is that epistatic interactions among mutations prevent the emergence of some mutations, or, in other words, two adaptive mutations may become deleterious when residing on the same genome. In line with this, the combination S:E484K/Q and S:P681H/R is not a common one, except in Omicron (albeit E484A) where over 30 S amino acid replacements have been observed. Indeed, several reports have suggested that abundant epistatic interactions led to the emergence of Omicron^46^, and these may allow the co-existence of S:E484A with S:P681H and others. It remains an enigma where Omicron first arose^47^, yet the fact that we see potentially epistatic interactions occurring in some chronic infections, coupled with the finding that Omicron is characterized by many such pairs of mutations, suggest that lengthy chronic infections may allow an exploration of the SARS-CoV-2 fitness landscape that may allow crossing ‘fitness valleys’ when the virus traverses through different organs and niches. Ultimately, in one of many chronic infections, a variant may emerge that both effectively evades antibodies and is also highly transmissible, as is the case of Omicron and, to some extent, also other VOCs.

### Limitations of this study

Although this study aggregated evidence from many isolated case reports, our sample size is still quite small and may also suffer from biases, such as the imbalanced age and sex structure of the sample and the wide variety of background conditions and treatments of the patients. For example, some studies selected patients with particular characteristics (for example, ref. ^48^), and different sequencing strategies and bioinformatics approaches were used to generate the data, leading, in some studies, to the possibility of batch effects (Methods and Supplementary Table 7). We also noted variation in the details of the clinical metadata reported across studies. Finally, we note that most cases analyzed herein were from chronic infections with variants that pre-dated the era of VOCs. With more variants emerging over time, many other changes have occurred as well: vaccinations were introduced, the level of global immunity has changed and marked behavioral changes have occurred, making future analyses even more complex. All in all, larger sample sizes of chronically infected patients with different variants across time, including dense sampling from different niches and organs, will be necessary to obtain a more robust and global view of intra-host evolution during chronic infections and the potential of such variants for infection and transmission.

To summarize, viral rebound can be viewed as a warning signal that a VOC-like mutation occurred in the patient, and extra caution may be warranted: genetic sequencing, isolation and close monitoring of contacts may be crucial for containment. More extensive monitoring and research of chronic infections is necessary to understand the precise factors determining when and if a variant generated in chronic infection becomes highly transmissible.

## Methods

### TASMC virus genome sequencing

Leftover RNA from nasopharyngeal swabs was obtained from six chronically infected individuals across several different timepoints for each patient (Supplementary Table 1). Sequences from patient 2 were available from our previous study^15^; patients 1 and 3 were also previously sequenced^14^, but here we added on additional timepoints. All patients were positive via COVID-19 RT–qPCR tests of SARS-CoV-2 for a period longer than 3 weeks, and all were immunocompromised. All relevant clinical data were collected and are summarized in Table 1 and Supplementary Tables 2 and 3. All samples underwent whole-genome SARS-CoV-2 sequencing using the V3 ARTIC protocol (https://artic.network/ncov-2019), and the samples were sequenced using Illumina MiSeq 250-cycle V2 kits at the Technion Genomic Center in Israel. This study was approved by the TASMC Helsinki committee (approval no. 1042-20-TLV) and by the Tel Aviv University institutional review board (approval no. 0004435-1), with an exemption from informed consent based on the use of retrospective fully anonymized samples and data.

### Determining genome consensus sequences and lineage determination

pTrimmer^55^ was used to trim the various primers that were used in the multiplex PCR. The raw reads were mapped to the reference genome of SARS-CoV-2 (GenBank ID NC_045512). Mapping and variant calling was performed using our AccuNGS pipeline^35^. The consensus sequence of each sample was determined based on sites with coverage of at least 10×. As we and others have observed, the sequencing process may lead to erroneous mutations being called, and this is particularly exacerbated at low VLs^10,11,35^. To this end, only mutations with a frequency of at least 80% were introduced into the consensus sequence (hereby referred to as substitutions or fixed mutations); loci with mixed populations at frequencies lower than 80% were considered ambiguous and marked by ‘N’.

For each patient, substitutions as compared to the reference that appeared in the first timepoint were marked as background substitutions and were not considered for any further analyses. Only substitutions that were added in the following timepoints were considered. The ‘Pangolin’ network was used to identify the consensus sequence lineage (https://cov-lineages.org/resources/pangolin.html)^56^. This allowed us to rule out re-infection in all patients but one: the last timepoint of patient 6 from day 48 of infection was inferred to be due to re-infection (B.1.1.7 as compared to B.1.1.50 in all earlier timepoints). The last timepoint from this patient was removed from the analysis, leaving a total of 37 days. Visual inspection of the results led to a suspected contamination at day 27 of patient 5, as several B.1.1.7 LDMs were observed on an otherwise B.1.1.50 background. This sample was omitted from the analysis.

### Re-analysis of previous case reports

Previously published case reports were selected by mining the literature for the keywords ‘prolonged’, ‘persistent’ or ‘chronic’ ‘SARS-CoV-2 infection’ up until 15 November 2021. Although some studies validated the chronic nature of infection by performing culturing of the virus or by measuring subgenomic RNA (sgRNA), several did not. Thus, when culture/sgRNA data were unavailable, we filtered out patients with infections of fewer than 20 days, based on a meta-analysis that showed that viral shedding was mostly limited to 20 days (see main text). We verified intermediate to high VL (Ct values of 27 or lower) at some time exceeding day 20 as a proxy for ensuring that shedding was not of residual non-infectious virus. Only studies that performed longitudinal whole-genome sequencing were maintained. In Tarhini et al.^53^, patient 3 was removed from the analysis as the authors identified a co-infection event. Sequencing data for Jensen et al.^48^ were obtained from the authors, albeit with missing data—the first timepoint for patient D was missing. We, thus, relied on the report by the authors on S:E484 at timepoint 1, and only mutation S:E484K was considered in timepoint 2 for patient D.

When re-analyzing the sequence data, we relied on the mutation frequencies reported in each publication, notably because raw data were unavailable in most of the papers. Although the methods used for mapping and base-calling somewhat differed among the different papers (Supplementary Table 7), in line with the above, we focused on mutations with a frequency higher than 80%, which was not present in the first timepoint sequenced. This led to a conservative approach that is likely less affected by the differences in the various publications’ bioinformatics approaches. When analyzing viral polymorphisms across time (Fig. 3), we also present mutations at lower frequencies, as reported by each individual publication, for illustrative purposes. All relevant clinical data were collected and are summarized in Table 1 and Supplementary Tables 2 and 3. Ct values for RT–qPCR tests were also extracted from all publications (Supplementary Table 6). A test was considered borderline if the Ct ranged from 34 to 37; a test was considered negative if it was reported as such or was equal to 38 or higher. In one study^48^, raw VL measurements were presented in terms of number of copies per milliliter of sample, and a negative result was reported when zero copies were detected.

### Statistics and reproducibility

As described above, this study was designed to compile all available viral genomic data of published SARS-CoV-2 chronically infected individuals. Hence, no statistical method was used to predetermine sample size; experiments were not randomized; and the investigators were not blinded to allocation during experiments and outcome assessment.

### Characterization of substitution events in chronic infections

A summary of all substitution events was compiled into three tables. Supplementary Table 1 lists every substitution and the patient(s) where it was found. Data on the proportion of a given substitution in the general global viral samples were taken from http://cov-glue-viz.cvr.gla.ac.uk/, and counts of substitutions along a sampled phylogeny of approximately 3,500 sequences were taken from NextStrain (https://nextstrain.org/)^21^ (Supplementary Table 9). Each of the S amino acid replacements was queried to see if information exists on antibody evasion for a given mutation and was marked as enhancing antibody evasion if a publication was available showing direct experimental evidence supporting this (Supplementary Table 1), which does not necessarily capture the full in vivo repertoire of antibody evasion. Moreover, we note that that could not directly test for antibody evasion in each patient but, rather, could infer it indirectly.

Supplementary Table 2 lists information (clinical and mutational, across all timepoints) per patient, whereas Supplementary Table 3 lists information per timepoint sequenced for each patient. When two samples from the same timepoint were available from different niches, we presented only the nasopharyngeal one, because our focus is primarily on this niche (with the exception of Fig. 3). Counts and information of substitutions per patient are listed in Supplementary Table 2 for both the S region and the rest of the genome. Transient substitutions (that re-appeared at a certain timepoint) were counted only once.

### Substitution events histogram

We counted the total number of substitutions observed at each position in the genome in four sets of sequences:

- Our set of chronic infections, with substitutions counted once at any timepoint sequenced.
- The LDM of the five VOCs (Alpha, Beta, Gamma, Beta and Omicron 21K, also known as BA.1), as defined by https://covariants.org, which relies on data from GISAID^57^. Substitutions shared by two or more VOCs due to shared ancestry were counted once.
- A subset of sequences from NextStrain (https://nextstrain.org/)^21^ (Supplementary Table 9). Sequences were filtered to those earlier than 16 August 2020. The numbers of events per genome position were downloaded from the site.
- A subset of sequences of the three globally dominant VOCs (Alpha, Delta and Omicron 21K) after their emergence, from NextStrain (Supplementary Table 9). We noted a large number of reversions among the Omicron sequences, mostly confined to the S region. We considered that these are most likely artifacts of low coverage coupled assemblers calling the reference sequence when a locus is not sequenced well, instead of assigning an ambiguous ‘N’. Accordingly, we masked the positions of LDMs across all the set of sequences in this subset.

To eliminate sequencing artifacts, all events that occurred in (c) or (d) in the first 130 bases or last 50 bases of the virus genome were trimmed off, as suggested in the NextStrain protocol^21^. Sites known to be associated with sequencing problems (https://virological.org/t/issues-with-sars-cov-2-sequencing-data/473) were also masked out.

All sequences used in the analyses for (c) and (d) are listed in Supplementary Table 9, and substitutions that are shown in Fig. 1 and Supplementary Fig. 1 are available in Supplementary Table 4.

A histogram was created for each of the three sets, with bin sizes of 500 bases. The probability of observing a given number of substitutions in a bin was calculated based on a binomial distribution with *p* = 500/*L* (*L* being the reference genome length), *n* being the sum of all substitutions observed in a set and *k* being the observed number of substitutions in a given bin. Correction for multiple testing was performed using the false discovery rate (FDR) of Benjamini–Hochberg^58^.

### Co-occurring substitutions

We began by identifying all pairs of substitutions that occurred in two or more of the patients with chronic infection. Fisher’s exact test was used to identify a deviation from expected individual frequencies. After this, we tested for co-occurrence of the set of enriched pairs in the global set of sequences by querying ViruSurf (http://geco.deib.polimi.it/virusurf/)^59^. Similarly to the described above, we also tested for enrichment of pairs of mutations, but the larger sample size of mutants permitted the use of a *χ*^2^ test to assess for deviation from expected individual frequencies by comparing marginal probabilities with paired probabilities (number of degrees of freedom was 1). All multiple testing was accounted for by FDR^58^.

### Machine learning approach for explaining antibody evasion

We compiled a dataset of 146 sequenced timepoints belonging to the 27 patients based on Supplementary Tables 2 and 3. Each timepoint was described using seven features: age, sex, days-since-infection, B cell depletion, steroid treatment, ABT and viral rebound, with the latter five features as binary features. The outcome was defined as whether or not an antibody evasion mutation was detected at the given timepoint. Severe B cell depletion was defined if one of the two following conditions was met: treatment with anti-B-cell-directed antibodies up to 9 months before COVID-19 detection or a blood test measurement of low B cell markers. Steroid treatment was defined if a dosage higher than 20 mg of prednisone per day (or equivalent for other drugs) was administered. ABT was defined as positive if a treatment of CP, hyperimmune plasma or mAbs were administered up to 7 days before the treatment. Finally, viral rebound was defined as positive if the COVID-19 PCR test just before the sequenced timepoint was borderline or negative (Supplementary Tables 2 and 3).

To account for the unique structure of these data and to avoid data leakage, we trained a random forest classifier with 500 trees, using ‘leave one patient out’ cross-validation: each split contained all timepoints of *n* − 1 patients as a train set, and the test set contained the remaining timepoints of the patient excluded from the train. Accuracy metrics, such as accuracy, precision, recall and F1 score, were calculated by weighting each metric score in each split by the number of observations in the test set, thus giving less weight to models tested on patients with a small number of timepoints.

After validating that the model is accurate enough (weighted F1 score of 0.78, weighted accuracy of 0.82), we used two approaches for assessing feature effect on the outcome of antibody evasion: the feature importance as calculated by the trees and SHAP values^31,32^. SHAP values were obtained for each mutation in each test set (that is, for each patient separately) (Fig. 1) and were aggregated to obtain the average effect that each feature has on the model’s outcome (Fig. 1 and Supplementary Figs. 2 and 3).

We also examined multiple classifiers, such as support vector machine, logistic regression and a simple decision tree, revealing that the results were highly robust to the type of classifier (Supplementary Figs. 2 and 3). Fitting the models and obtaining SHAP values were performed using Python 3.8 and the packages Scikit-learn version 0.24.1 and SHAP version 0.37.

### Reporting summary

Further information on research design is available in the Nature Research Reporting Summary linked to this article.

## Online content

Any methods, additional references, Nature Research reporting summaries, source data, extended data, supplementary information, acknowledgements, peer review information; details of author contributions and competing interests; and statements of data and code availability are available at 10.1038/s41591-022-01882-4.

## Supplementary information

Supplementary Information Supplementary Figs. 1–3

Reporting Summary

Supplementary Tables 1–9 1. Summary of all mutations fixed in chronic infections; 2. Supplementary information per patients with chronic infections; 3. Timepoints sequenced per patients with chronic infections; 4. Mutation counts used to create Fig. 1 and Supplementary Fig. 1; 5. Details of virus isolates sequenced in this paper; 6. Information supporting replicative virus across chronic infections; 7. Information on bioinformatics procedures used in each supporting publication; 8. *P* value summary for Fig. 1a and Supplementary Fig. 1; 9. Supplemental acknowledgments GISAID

## Data Availability

All consensus sequences for the patients sequenced herein were deposited in GISAID, and raw sequencing reads were uploaded to the National Center of Biotechnology Information Sequence Read Archive under accession number PRJNA803960 (Supplementary Table 5).

## Code availability

Code, input data and documentation of the machine learning models are available via GitHub (https://github.com/SternLabTAU/chronicSC2).
