# Supplementary material for: Drivers of adaptive evolution during chronic SARS-CoV-2 infections
Source: Nat Med. 2022 Jun 20;28(7):1501–8. doi: 10.1038/s41591-022-01882-4 (PMC9307477; doi:10.1038/s41591-022-01882-4)
Supplement: Supplementary file 1 — Supplementary Figs. 1–3 [file 41591_2022_1882_MOESM1_ESM.pdf]

nature portfolio

https://doi.org/10.1038/s41591-022-01882-4

Supplementary information

Drivers of adaptive evolution during chronic SARS-CoV-2 infections

In the format provided by the authors and unedited

Supplementary figures for Drivers of adaptive evolution during chronic SARS-CoV-2 infections by Harari et al.

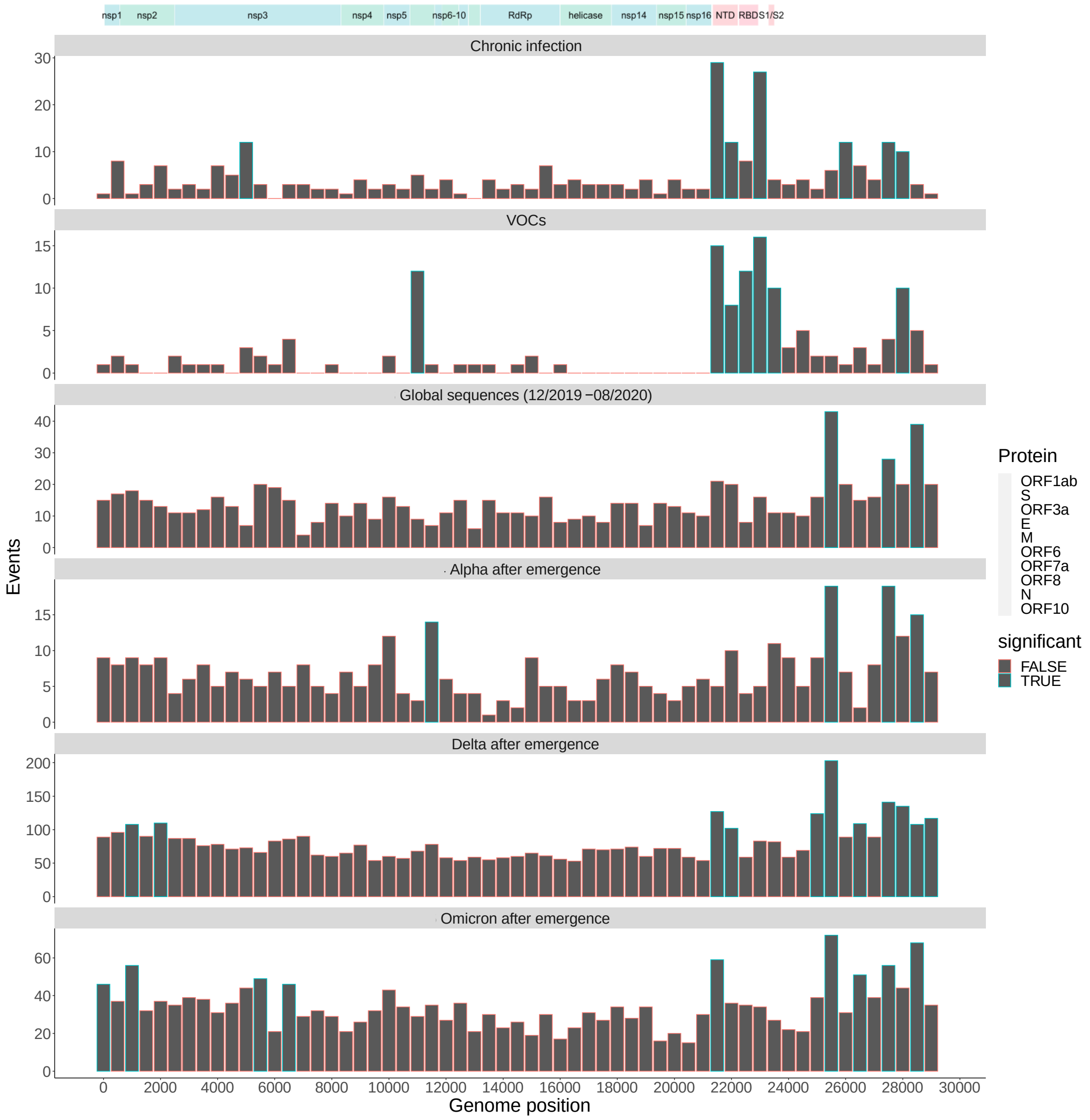

Figure S1. Comparison of substitutions observed in chronic patients and other globally observed patterns of substitutions. The first three panels are identical to those in Fig. 1A, with all details on binning and statistical analyses identical to those shown in Fig. 1A. In addition, the bottom three panels display substitutions observed post-VOC emergence, for the three globally dispersed VOCs Alpha, Delta and Omicron (BA.1), based on the sample of sequences available in the global build of NextStrain (accessed June 1, 2022). We noted that between 50% and 67% of substitutions were non-synonymous in post- Alpha, Delta, and Omicron sequences, similar to the value of 61% observed during the first nine months of viral circulation, and much lower than the values observed for chronic infections and VOCs (see main text). This supports the notion that post-VOC emergence, during

spread along acute infection transmission chains, the tight transmission bottleneck strongly decreases selection and leads to a mostly neutral pattern of mutations. Overall, the patterns of mutations observed along the genome are similar in the two upper panels (chronic infections and VOCs), and in the four lower panels (global sequences of the early pandemic, and post-emergence for Alpha, Delta and Omicron). Notably, we do see some enrichment for mutations at the beginning of the Spike protein in post-emergence Delta and Omicron, which may signify selection. These residues belong to the "NTD supersite" and are associated with immune evasion [1,2], yet the NTD is also associated with structural rearrangements following ACE2 receptor binding [3].

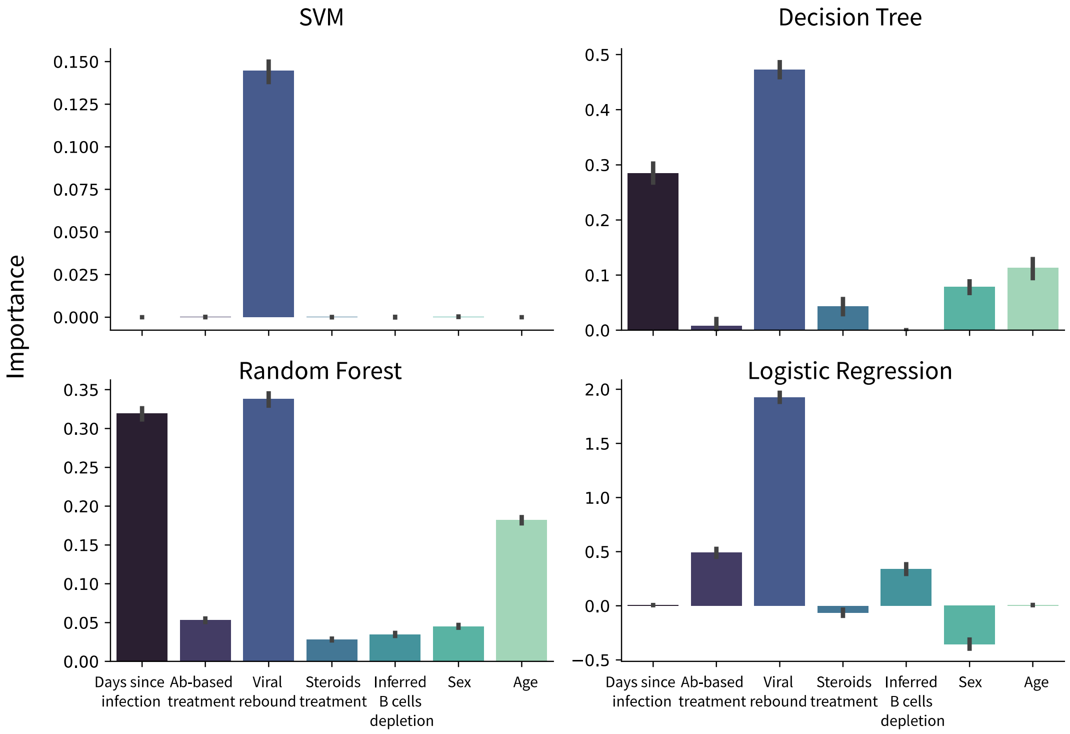

Figure S2. Feature importance by fitted model. The importance of each feature by model type for 27 chronically infected patients as obtained by cross-validation. For SVM (f1 score 0.74, accuracy 0.81) and logistic regression (f1 score 0.77, accuracy 0.82) models, the importance is obtained by the weights learned by the classifier and assigned to each feature (also referred to as coefficients). For the tree-based models, decision tree (f1 score 0.78, accuracy 0.80) and random forest (f1 score 0.78, accuracy 0.82), the importance is defined as the gini impurity-based importance (mean decrease in impurity). Data are presented as mean values +/- SD, obtained for n=27 independent patients.

Decision Tree SHAP

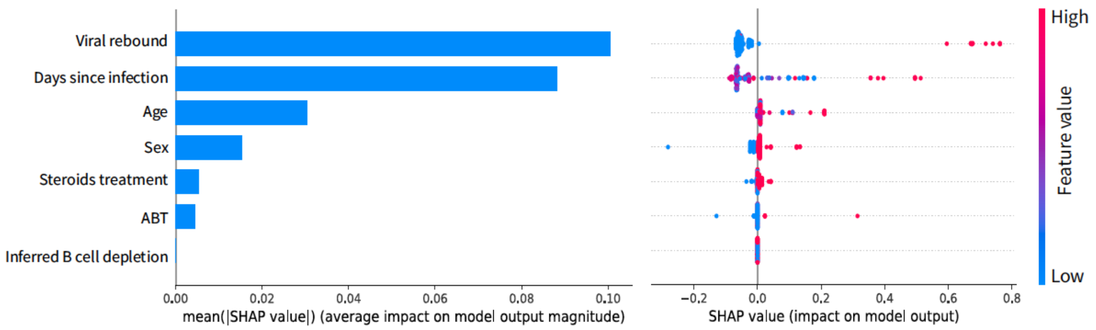

Figure S3. SHAP values for the decision tree classifier. The bars show the average impact of each feature on antibody-evasion, shown as mean absolute SHAP values, ordered based on contribution. For each bar the individual SHAP values for each feature are presented. The color range corresponds to the individual's feature value, from red (high value) to blue (low value).

References

1 Harvey, W. T. *et al.* SARS-CoV-2 variants, spike mutations and immune escape. *Nat Rev Microbiol* **19**, 409-424, doi:10.1038/s41579-021-00573-0 (2021).

2 Lok, S. M. An NTD supersite of attack. *Cell host & microbe* **29**, 744-746, doi:10.1016/j.chom.2021.04.010 (2021).

3 Benton, D. J. *et al.* Receptor binding and priming of the spike protein of SARS-CoV-2 for membrane fusion. *Nature* **588**, 327-330, doi:10.1038/s41586-020-2772-0 (2020).
