# Supplementary material for: Drivers of adaptive evolution during chronic SARS-CoV-2 infections
Source: Nat Med. 2022 Jun 20;28(7):1501–8. doi: 10.1038/s41591-022-01882-4 (PMC9307477; doi:10.1038/s41591-022-01882-4)
Supplement: Supplementary file 2 — Reporting Summary [file 41591_2022_1882_MOESM2_ESM.pdf]

nature portfolio

Corresponding author(s): Adi Stern
Last updated by author(s): May 19, 2022

Reporting Summary

Nature Portfolio wishes to improve the reproducibility of the work that we publish. This form provides structure for consistency and transparency in reporting. For further information on Nature Portfolio policies, see our Editorial Policies and the Editorial Policy Checklist.

Statistics

For all statistical analyses, confirm that the following items are present in the figure legend, table legend, main text, or Methods section.

n/a	Confirmed	
☐	☒	The exact sample size (*n*) for each experimental group/condition, given as a discrete number and unit of measurement
☐	☒	A statement on whether measurements were taken from distinct samples or whether the same sample was measured repeatedly
☐	☒	The statistical test(s) used AND whether they are one- or two-sided<br>*Only common tests should be described solely by name; describe more complex techniques in the Methods section.*
☐	☒	A description of all covariates tested
☐	☒	A description of any assumptions or corrections, such as tests of normality and adjustment for multiple comparisons
☐	☒	A full description of the statistical parameters including central tendency (e.g. means) or other basic estimates (e.g. regression coefficient) AND variation (e.g. standard deviation) or associated estimates of uncertainty (e.g. confidence intervals)
☐	☒	For null hypothesis testing, the test statistic (e.g. *F*, *t*, *r*) with confidence intervals, effect sizes, degrees of freedom and *P* value noted<br>*Give P values as exact values whenever suitable.*
☒	☐	For Bayesian analysis, information on the choice of priors and Markov chain Monte Carlo settings
☒	☐	For hierarchical and complex designs, identification of the appropriate level for tests and full reporting of outcomes
☒	☐	Estimates of effect sizes (e.g. Cohen's *d*, Pearson's *r*), indicating how they were calculated

Our web collection on statistics for biologists contains articles on many of the points above.

Software and code

Policy information about availability of computer code

Data collection	None
Data analysis	pTrimmer v1.3.1, AccuNGS V1, pangolin v3.1.19, R software version 4.03, Python > v3.7.4, Pandas v0.24.2, ggplot v3.3.33, SciPy v1.5.4, Matplotlib v3.2.1, Numpy v1.19.5, Scikit-learn version 0.24.1 and shap version 0.37. Custom code used in this study is available in the following link: https://github.com/SternLabTAU/chronicSC2.

For manuscripts utilizing custom algorithms or software that are central to the research but not yet described in published literature, software must be made available to editors and reviewers. We strongly encourage code deposition in a community repository (e.g. GitHub). See the Nature Portfolio guidelines for submitting code & software for further information.

Data

Policy information about availability of data

All manuscripts must include a data availability statement. This statement should provide the following information, where applicable:

- Accession codes, unique identifiers, or web links for publicly available datasets
- A description of any restrictions on data availability
- For clinical datasets or third party data, please ensure that the statement adheres to our policy

Data that support the findings of this study have been deposit to relevant databases: viral sequences of all patients that were sequenced herein are available in GISAID (https://www.gisaid.org/) and compatible accession numbers denoted in Table S5. All raw sequencing data are available in NCBI sequence read archive (https://submit.ncbi.nlm.nih.gov/) submission number PRJNA803960. The reference genome of SARS-CoV-2 used herein is available in GenBank (https://www.ncbi.nlm.nih.gov/genbank/) under the the accession number NC_045512. For the globally circulating genomes analysis, data were obtained from Nextstrain (https://nextstrain.org/ncov/gisaid/global), Table S9 lists the accession numbers of sequenced used for this analysis.

Field-specific reporting

Please select the one below that is the best fit for your research. If you are not sure, read the appropriate sections before making your selection.

☐ Life sciences ☐ Behavioural & social sciences ☒ Ecological, evolutionary & environmental sciences

For a reference copy of the document with all sections, see nature.com/documents/nr-reporting-summary-flat.pdf

Ecological, evolutionary & environmental sciences study design

All studies must disclose on these points even when the disclosure is negative.

Study description	This study aimed at charactering and comparing mutations that accumulated during SARS-CoV-2 chronic infections. This was performed by re-analyzing previously published case reports, and by sequencing viral genomes from six such patients herein.
Research sample	We focused on all SARS-CoV-2 chronically infected patients that were admitted to Sourasky Medical Center, Tel Aviv, Israel during the period of the study (March 2020 through April 2021). Viral RNA was sequenced from leftovers of the patients samples at all available time-points. All patients and samples information are available in Tables 1, S2 and S6. In parallel, all published case reports of chronically infected patients were mined for sequencing data. Thus, overall, this study aimed at obtaining the maximal number of chronic infections with relevant sequencing data .
Sampling strategy	All cases of patients with immune deficiency and viral RNA shedding of at least 20 days, with Ct<=27 at a time-point exceeding day 20. The number of patients that were sequenced herein is n=6, with n=21 additional previously published patients, i.e. the total number of patients analyzed in this study is 27. This is the largest dataset of chronic SARS-CoV-2 patients that has been analyzed to date.
Data collection	Extracted RNA was collected in Tel-Aviv Sourasky Medical Center, and sent for sequencing in the Genomic center in the Technion (Haifa, Israel). Background data regarding the patient status was recorded by the patient's attending physician (Sourasky Medical Center) on a computer, including: sex, age range, background medical condition, treatment information, sample collection type and dates, and Ct values. This information was also collected for all case reports that were considered for the re-analysis (information available in Tables S2,S3,S5,S6)
Timing and spatial scale	Samples in this manuscript were retrospectively collected from March 2020 to April 2021 at Sourasky Medical Center, Tel Aviv, Israel .
Data exclusions	We included patients displaying high viral load shedding for 20 or more days, who also had some evidence for an immune-suppressed background; we hence excluded any samples that did not adhere to the latter criteria.
Reproducibility	This dataset is unique and therefore is not reproducible.
Randomization	In this study we analyzed a specific and unique type of patients, therefore all relevant cases were considered for this analysis and randomization was not relevant. Our focus was on maximizing the number of chronic infections analyzed.
Blinding	Blinding was not performed since it was not relevant - as described above under Randomization. Only general data per patient (described above) was available to the researchers to ensure privacy of patients.

Did the study involve field work? ☐ Yes ☒ No

Reporting for specific materials, systems and methods

We require information from authors about some types of materials, experimental systems and methods used in many studies. Here, indicate whether each material, system or method listed is relevant to your study. If you are not sure if a list item applies to your research, read the appropriate section before selecting a response.

Materials & experimental systems

n/a	Involved in the study	
☒	☐	Antibodies
☒	☐	Eukaryotic cell lines
☒	☐	Palaeontology and archaeology
☒	☐	Animals and other organisms
☐	☒	Human research participants
☒	☐	Clinical data
☒	☐	Dual use research of concern

Methods

n/a	Involved in the study	
☒	☐	ChIP-seq
☒	☐	Flow cytometry
☒	☐	MRI-based neuroimaging

Human research participants

Policy information about studies involving human research participants

Population characteristics	Patients (of all age, sex and race) with a chronic SARS-CoV-2 infection (as defined above in sampling strategy) were selected for this study: mean age (standard deviation) 55 (21.3) years; 17/27 male.
Recruitment	No specific recruitment was performed since this is a retrospective cohort. All samples were collected from leftovers of routine sampling during the patient's admission.
Ethics oversight	This study was approved by the Tel Aviv Sourasky Medical Center Helsinki committee (approval No. 1042-20-TLV), and by the Tel Aviv University IRB (approval number 0004435-1).

Note that full information on the approval of the study protocol must also be provided in the manuscript.
